# Comparative Population Assessments of *Nautilus* sp. in the Philippines, Australia, Fiji, and American Samoa Using Baited Remote Underwater Video Systems

**DOI:** 10.1371/journal.pone.0100799

**Published:** 2014-06-23

**Authors:** Gregory J. Barord, Frederick Dooley, Andrew Dunstan, Anthony Ilano, Karen N. Keister, Heike Neumeister, Thomas Preuss, Shane Schoepfer, Peter D. Ward

**Affiliations:** 1 Department of Biology, Graduate Center, City University of New York, New York, New York, United States of America; 2 Department of Biology, Brooklyn College, City University of New York, Brooklyn, New York, United States of America; 3 Department of Biology, University of Washington, Seattle, Washington, United States of America; 4 School of Biomedical Sciences, University of Queensland, Brisbane St. Lucia, Queensland, Australia; 5 Department of Biology, University of San Carlos, Cebu City, Cebu, Philippines; 6 Department of Biology, Alaskan Observers Incorporated, Seattle, Washington, United States of America; 7 Department of Psychology, Hunter College, City University of New York, New York, New York, United States of America; 8 Department of Earth and Space Sciences, University of Washington, Seattle, Washington, United States of America; Seagrass Ecosystem Research Group, Swansea University, United Kingdom

## Abstract

The extant species of *Nautilus* and *Allonautilus* (Cephalopoda) inhabit fore-reef slope environments across a large geographic area of the tropical western Pacific and eastern Indian Oceans. While many aspects of their biology and behavior are now well-documented, uncertainties concerning their current populations and ecological role in the deeper, fore-reef slope environments remain. Given the historical to current day presence of nautilus fisheries at various locales across the Pacific and Indian Oceans, a comparative assessment of the current state of nautilus populations is critical to determine whether conservation measures are warranted. We used baited remote underwater video systems (BRUVS) to make quantitative photographic records as a means of estimating population abundance of *Nautilus* sp. at sites in the Philippine Islands, American Samoa, Fiji, and along an approximately 125 km transect on the fore reef slope of the Great Barrier Reef from east of Cairns to east of Lizard Island, Australia. Each site was selected based on its geography, historical abundance, and the presence (Philippines) or absence (other sites) of *Nautilus* fisheries The results from these observations indicate that there are significantly fewer nautiluses observable with this method in the Philippine Islands site. While there may be multiple possibilities for this difference, the most parsimonious is that the Philippine Islands population has been reduced due to fishing. When compared to historical trap records from the same site the data suggest there have been far more nautiluses at this site in the past. The BRUVS proved to be a valuable tool to measure *Nautilus* abundance in the deep sea (300–400 m) while reducing our overall footprint on the environment.

## Introduction

Nautiluses are part of an ancient nautiloid lineage that has existed for nearly 500 million years [1]. All living nautiluses inhabit deep coral reef slopes throughout the Indo-Pacific and comprise two genera: *Nautilus* and *Allonautilus* [2], [3], [4]. Their habitat is constrained by depth implosion limits of 800 m [5], surface temperature limits of 25°C [6] and a nektobethic life style [7], living in close association with reef slopes and ocean floors rather than in the mid-water or surface waters. These limitations effectively isolate local populations of nautiluses and may restrict gene flow, producing genetically distinct populations [8], [9], [10]. This also limits the possibility of re-colonization events if local populations become depleted. Recent genetic work suggests the possibility that nautiluses have been undergoing a rapid adaptive radiation since the Pliocene, and as such, there may be tens (or more) of currently unrecognized but separate sibling species unique to islands and land masses separated by water depths greater than the 800 m deep implosion depth [11]. Thus, our best understanding of the genetic makeup of the various species is that the loss of any population results in an overall loss of genetic biodiversity.

Nautiluses can be captured using baited traps, which they can locate using chemoreception from significant distances. The ease of their capture using these traps coupled with the value of their shells, in both the shell and jewelry trade, have led to small and large scale fisheries in the Philippines, New Caledonia, and perhaps Indonesia in the 1970s onward [12], [13], [14]. However, lack of monitoring has obscured any objective understanding of either the size or biological effects of these fisheries on the standing populations in fished locales.

Nautiluses have a reproductive strategy typical of many deep water animals in showing slow growth to maturity (in this case, 12–15 years [15], [16], [17]), low fecundity (0–10 eggs [18], [19]), and long developmental times (1 year [18], [19]). As in the many fore reef slope fisheries of fish and invertebrates with similar life history strategies, such as the deep-water fish *Etelis*, Orange Roughy, and various deeper water, larger crabs such as *Geryon*, all of which have experienced documented population declines in specific localities where they have been heavily fished. These characteristics suggest that nautilus populations are inherently vulnerable to exploitation and may exponentially compound the effects of fisheries in reducing new recruitment. Yet to date, there have been only anecdotal reports describing population declines in two traditional nautilus fishing grounds (both in the Philippine Islands: Bohol Sea and Tanon Strait). While our own field observations have confirmed that nautilus fishing continues in the Bohol Sea, the Tanon Strait population may have already crashed by 1987 [13], [20]. Yet, new evidence indicates that large numbers of nautiluses continue to be killed for their shells based on the first ever report of the nautilus shell trade by the United States Fish and Wildlife Service [21]. From 2006–2010, the number of nautilus shells or shell products (such as jewelry) imported into the United States exceeded 500,000 items. As a large number of these items were individual shells, these numbers attest to the effectiveness and scope of the global nautilus fishery.

The “normal” population density of distinct populations of either *Nautilus* (with four currently accepted species: *N. pompilius, N. stenomphalus, N. macromphalus* and *N. belauensis*) or *Allonautilus* (with two: *A. scrobiculatus* and *A. perforatus*) remained unknown until 2011 [22]. Based on the large catches per trap from virtually all known *Nautilus* and *Allonautilus* trapping efforts, where as many as 60 nautiluses can be recovered from a single, 1 m^3^ trap deployed for a single night [6], it has been assumed that nautiluses are relatively common. However, new information has demonstrated that they are superbly adapted for discovering food from great geographic distances [23], leading to the alternative possibility that the large catches are misleading with regard to actual population numbers. With only one current estimate of a nautilus population available [22] and a survey-based study suggesting up to 80% declines in catch per unit effort in locations across the Philippines [24], it was imperative to assess additional populations of nautiluses.

Baited remote underwater video systems (BRUVS) are a relatively new method of population assessment in marine environments [25], [26], [27], [28]. The majority of these studies were designed for use in relatively shallow coral reef systems. The use of BRUVS in the deep sea, however, has not been consistently researched. Although BRUVS have their own inherent biases, specifically related to the use of bait [29], the use of BRUVS as a preferred alternative over other methods because it is less destructive [30] and can provide estimates of relative abundance of economically important species [31]. When assessing unknown populations that are assumed to be under threat, such as nautiluses, BRUVS are also non-destructive and do not remove individuals from the population [32]. Here, we used BRUVS to collect data from four previously un-sampled populations of nautiluses and provide the first quantification of the effect of fisheries on nautilus populations.

## Results and Discussion

**Figure 1:**
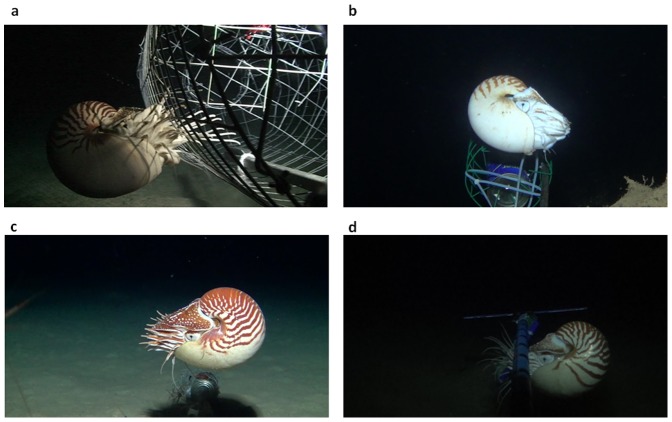
a–d. Photographic identification of nautiluses at each location. Photographs of nautiluses taken from the underwater video footage from Australia (1a), Fiji (1b), American Samoa (1c), and the Philippines (1d).

We used baited remote underwater video systems (BRUVS) in 2011–2013 to make quantitative measurements of the population abundance of nautiluses attracted to this system at four geographic locations in the Indo-Pacific: the Panglao region of the Bohol Sea, Philippines; the Great Barrier Reef along a transect from Cairns to Lizard Island; the Beqa Passage in Viti Levu, Fiji; and Taena Bank near Pago Pago harbor, American Samoa. From the video footage (see Video S1, S2, S3, and S4) we identified individual nautiluses using photographic identification of each specimen (Fig. 1a–d) through image recognition software [33] as the individual color patterns of nautiluses are unique. From these data we have calculated population abundance data at each geographic location (Table 1).

**Table 1:** Population abundance values of the each location sampled including prior data from Osprey Reef, Australia^10^ representing all currently sampled *Nautilus* populations.

Location	Number of Nautiluses	*Nautilus* Attraction Rate (N/hr)	Population Abundance (N/km2)
Osprey Reef, Australia	68	4.03	13.60
Great Barrier Reef, Australia	92	0.60	0.34
Beqa Passage, Fiji	20	0.79	0.21
Taena Bank, American Samoa	22	0.51	0.16
Bohol Sea, Philippines	6	0.09*	0.03*

Even with our new observations from additional targeted observation sites, the largest number of nautiluses observed was measured from Osprey Reef and the Great Barrier Reef locations in Australia (93 total/2.01 per km^−1^). Lesser numbers came from Beqa Passage, Fiji (20 total/1.58 per km^−1^), followed by Taena Bank, American Samoa (22/1.48 per km^−1^). The lowest numbers of all (6/0.25 per km^−1^), by far, were measured at the Panglao locality in the Bohol Sea, Philippines. Comparison between sites using paired t-test and linear regression demonstrate a highly significant (f = 9.99; df = 44; P<0.001) difference between the Philippines site and the other four non-fished sites (vs. Australia t = 22.2; Fiji t = 7.42; A. Samoa t = 11.18; all P<0.001). Likewise, the attraction rates measured were greatest in the two Australian populations and lowest in the Philippines population, which was again significantly different than each of the non-fished sites (P<0.001).

Next, we used the data from above (number of nautiluses and attraction rates) to calculate a population abundance at each location. The population abundance values mirrored the total number of nautiluses and attraction rates measured at each site with the Philippines site being significantly different than the non-fished sites (P<0.001), while the non-fished sites were not significantly different from each other. We also believe that the population measures reported here might, in fact, be overestimates at each site, given the ability of nautiluses to locate food across long distances as well as their confined (depth dictated) habitats. Thus, natural populations may be more dispersed and representative of lower levels of abundances and densities.

The use of BRUVS as an estimator certainly provides new information useful in evaluating the possible effects of fishing or other environmental change. Yet it is very clear that many variables are at play in determining the number of nautiluses attracted to the baited traps, with the rate, directionality, and other factors affecting the concentration of chemical scent moving out into the surrounded waters virtually impossible to quantify [29], [30]. Thus, the use of BRUVS alone has its limitations. On the other hand, the results obtained here are consistent with a conclusion that the fished, Philippines populations are significantly different in not only the numbers of nautiluses attracted to the bait each night, but also in terms of the age-class structure of the attracted animals. For example the number of fully mature animals seen at Osprey Reef exceeded 80% [22], and this number is consistent with other studies of age class structure of sampled nautilus populations from Palau in the 1980 s [34], [35]. Our work showed that less than 50% of the observed animals in the Philippines are mature.

While the differences in population abundance observed here might be artifacts of the methodology, or, if real, related to factors other than fishing [21] (such as habitat change from increasing bottom temperatures, decreasing oxygen values, reduced food sources, and increased sedimentation), the presence of human fishing remains the most parsimonious explanation for smaller number of observed nautiluses in the Bohol region, and is the best explanation for what appears to be a complete abandonment of the once vigorous Tanon Strait nautilus fishery (the latter being geographically separated from the Bohol population). The fact that the latter population has not subsequently rebounded to a point where fishing has begun anew is certainly a red flag about that ability of nautilus populations to rebound even if all fishing were banned.

## Conclusions

The greatest surprise of our data was the uniformly low population sizes among nautilus populations attracted to BRUVS at both the fished and non-fished sites. These low numbers suggest that extant nautilid species are vulnerable to unregulated (or perhaps even regulated) fisheries and may also be affected by other environmental changes in the deep sea marine ecosystem, of which even less is known than nautilus populations. It may be that factors other than direct nautilus fishing are, or soon will affect not just nautiluses, but other species of the still poorly-known but large fore-reef slope communities and their environments of the tropical Indo-Pacific. Irrespective of this, these data provide valuable baseline information for future studies assessing fishery and/or environmental changes related to nautiluses and the flora and fauna of these deep sea habitats. We believe we have addressed significant gaps that have previously hindered regulatory and conservation agencies [21] and the results reported here appear to validate older historical claims of nautilus population collapse due to the global nautilus shell trade, and argue strongly for immediate international regulation of the *Nautilus* and *Allonautilus* shell trade.

## Materials and Methods

### Ethics Statement

This study did not involve endangered or protected species and no animals were collected. Research in the Philippines conducted in collaboration with University of San Carlos and no permit required as no animals were collected. Research in Australia conducted under permit from the Great Barrier Reef Marine Park Authority and the University of Queensland Animal Ethics Committee. Research in Fiji conducted under permit from the Department of Fisheries. Research in American Samoa conducted under permit from the Department of Marine and Wildlife Resources.

### Location

The study took place across four geographic locations in the Indo-Pacific with known nautilus populations. One fished population in the Philippines was compared to three non-fished populations in Australia, Fiji, and American Samoa. The fished population in the Philippines was located in the Bohol Sea off the coast of Panglao, Philippines (9°35′ 18.87″ N, 123°43′ 44.54″ E). The three non-fished populations were along a transect of the Great Barrier Reef from Cairns to Lizard Island (16°37′ 28.91″ S, 145°53′ 07.35″ E), Beqa Harbour near Pacific Harbour, Fiji (18°19′ 40.24″ S, 178°06′ 30.86″ E), and Taena Bank near the harbor of Pago Pago, American Samoa (14°19′ 19.57″ S, 170°38′ 57.78″ W).

### Data Collection

At each location, baited remote underwater video systems (BRUVS) were deployed to record the number of nautiluses attracted to bait over time. Each BRUVS unit consisted of a steel frame (100 cm×60 cm×75 cm), a Sony HD video camera in an underwater housing (Raytech Services PTY LTD), a white LED light source in an underwater housing, and a bait stick extending beyond the frame in view of the camera. While chicken meat was the primary bait used throughout the project (exclusively in the Philippines and Australia), limited resources in Fiji and American Samoa required the use of additional bait sources of tuna and mackerel when chicken was not available. Each BRUVS recorded up to 12 hours of video footage. The BRUVS were deployed at dusk (∼1800 hours) and were retrieved the following morning (∼0600 hours). The average deployment (soak) time was 12 hours. A total of three BRUVS systems were deployed at each site in Australia and spaced out 1 km from each other; the three BRUVS were not considered independent replicates for our analyses and were combined as one sample. The BRUVS were deployed in the Philippines, Fiji, and American Samoa using similar methods as Australia, the primary difference being that a total of two BRUVS were used instead of three for each night. Before deploying the BRUVS, a depth sounder was used to determine deployment depth. Although average depth between sites was ∼350 m, deployment depths ranged between 300 and 400 m depending upon the location and topography of the ocean floor. A GPS unit was used to record the position of each deployment as well as the retrieval.

The BRUVS were deployed at each site multiple times across several days. The number of BRUVS deployments at each location varied due to adverse weather conditions and changing resources in the field. A total of four successful deployment days was achieved in the Philippines and American Samoa; three successful deployment days were achieved in Fiji; and a total of six successful deployment days were achieved in Australia across three sites; two deployment days at each site (site 1: 15°59'52.80"S, 145°51'15.66"E; site 2: 15°30'38.82"S, 145°49'2.40"E; site 3: 15°50'36.99"S, 145°48'45.42"E). As the Australian population inhabited a barrier reef, sampling multiple locations along the reef provided the most appropriate data. These data were then combined together to determine an average value for each population measurement, at each site.

### Data Analysis

Each of the videos was reviewed and individual nautiluses were identified by their unique color pattern using the species recognition program, Hotspotter [33]. The population density of each sampled area was calculated using footage from the BRUVS units. The total number of nautiluses was recorded from each of the videos. Next, the speed of the nautiluses in the video was recorded using a known frame of reference. The speeds were averaged at the sites to determine an average speed of 0.10 m s^−1^ (or 360 km hr^−1^). These calculations are within range of several other swimming speed calculations in the literature [22], [36], [37]. This average speed was multiplied by the total length of the video to determine the maximum distance the nautilus could travel to reach the recording site. This maximum distance value was then inserted into a formula (area of a circle) as the radius to calculate the possible sampling area. Finally, the total number of nautiluses was divided by the sampling area to determine the population abundance of the area sampled. Statistical analysis was computed in R (R version 2.14.2). Means of populations and number collected by hours of observation were compared against each other using a paired T-Test. Secondly; the data was analyzed using a general linear regression model with ANOVA.

## Supporting Information

Video S1 **Video footage of BRUVS in Australia.** Here, our baited remote underwater video systems recorded several *Nautilus* attracted to our bait source.
(MP4)

Video S2 **Video footage of BRUVS in Fiji.** Here, our baited remote underwater video systems recorded one *Nautilus* as well as a shrimp in the foreground.
(MP4)

Video S3 **Video footage of BRUVS in American Samoa.** Here, our baited remote underwater video systems recorded two larger adult *Nautilus* and one juvenile *Nautilus*.
(MP4)

Video S4 **Video footage of BRUVS in the Philippines.** Here our baited remote underwater video systems record one *Nautilus* attracted to our bait as well as other species that that co-exist with *Nautilus*, such as the hagfish and shrimps.
(MP4)
