# Correction: Comparative Population Assessments of *Nautilus* sp. in the Philippines, Australia, Fiji, and American Samoa Using Baited Remote Underwater Video Systems

**DOI:** 10.1371/journal.pone.0107836

**Published:** 2014-09-08

**Authors:** The PLOS ONE Staff

In the Funding section, the funder Tiffany Foundation should be listed as The Tiffany & Co. Foundation.
